# Comparative Study of Popular Deep Learning Models for Machining Roughness Classification Using Sound and Force Signals

**DOI:** 10.3390/mi12121484

**Published:** 2021-11-29

**Authors:** Binayak Bhandari

**Affiliations:** Department of Railroad Engineering & Transport Management, Woosong University, Daejeon 300718, Korea; binayak@sis.ac.kr

**Keywords:** sound feature extraction, precision machining, Deep Learning, CNN, LSTM, MLP, attention mechanisms, Smart Factory, classification, confusion matrix

## Abstract

This study compared popular Deep Learning (DL) architectures to classify machining surface roughness using sound and force data. The DL architectures considered in this study include Multi-Layer Perceptron (MLP), Convolution Neural Network (CNN), Long Short-Term Memory (LSTM), and transformer. The classification was performed on the sound and force data generated during machining aluminum sheets for different levels of spindle speed, feed rate, depth of cut, and end-mill diameter, and it was trained on 30 s machining data (10–40 s) of the machining experiments. Since a raw audio waveform is seldom used in DL models, Mel-Spectrogram and Mel Frequency Cepstral Coefficients (MFCCs) audio feature extraction techniques were used in the DL models. The results of DL models were compared for the training–validation accuracy, training epochs, and training parameters of each model. Although the roughness classification by all the DL models was satisfactory (except for CNN with Mel-Spectrogram), the transformer-based modes had the highest training (>96%) and validation accuracies (≈90%). The CNN model with Mel-Spectrogram exhibited the worst training and inference accuracy, which is influenced by limited training data. Confusion matrices were plotted to observe the classification accuracy visually. The confusion matrices showed that the transformer model trained on Mel-Spectrogram and the transformer model trained on MFCCs correctly predicted 366 (or 91.5%) and 371 (or 92.7%) out of 400 test samples. This study also highlights the suitability and superiority of the transformer model for time series sound and force data and over other DL models.

## 1. Introduction

The Fourth Industrial Revolution, or Industry 4.0, represents a paradigm shift in the manufacturing industries through the introduction of intelligent manufacturing systems by integrating the Internet of Things (IoT), big data, and artificial intelligence (AI). Industries with such intelligent systems are called ‘smart factories’ [1,2,3]. The concept of the Smart Factory (SF) has been coined to define intelligent and digitized manufacturing. Digitization, intelligence, integration, the generation of engineering knowledge, and connectivity to Man, Machine, Material, Method, and Environment (4M&1E) make up the foundation of a Smart Factory [4]. Although large enterprises often implement advanced technology quickly in order to improve productivity and quality with the motive to maintain their dominance in the global market, small and medium-sized enterprises (SMEs) have difficulty adopting smart technologies because of financial and technical constraints.

**Figure 1:**
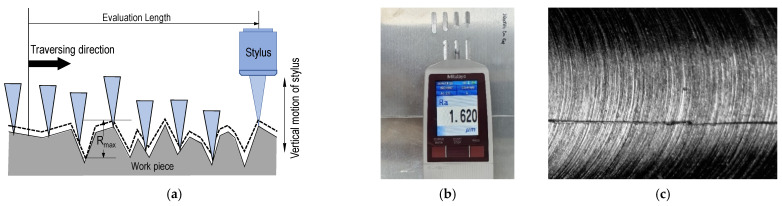
Roughness measurement: (**a**) stylus surface assessment illustration, (**b**) surface roughness tester in action, and (**c**) visible scratch after surface roughness measured using a stylus.

Milling is one of the standard machining methods widely adopted for machining metallic materials by precision manufacturing companies. Milling operation makes use of cutters with specific machining parameters to remove material from the workpiece while obtaining dimensional accuracy and high surface quality. In milled products, surface roughness is the crucial quality measurement often employed by an off-line method. Hence, modern machining enterprises utilizes Computer Numerical Control (CNC) machines to produce workpieces with fine details and tight tolerance. Machined parts with surface roughness within the threshold limit are accepted, but those outside the limit are discarded. The surface roughness of machined parts is measured through a profiling technique, using either a stylus or contactless laser-based methods. Most SMEs make use of the former technique because it is cheaper, simpler, and often more accurate. However, the stylus method is infamous for causing scratches on the sample surface because of measuring pressure, as shown in Figure 1.

Additionally, it is well known that the quality of a surface in end-milling operation depends on factors such as the spindle speed, feed rate, depth-of-cut, machining time, lubrication, cooling methods, and the work materials. The dimensional accuracy and surface roughness are the two most significant machining quality characteristics. To achieve a fine machining surface finish, it is essential to set the process parameters before machining operations [5]. Historically, engineers and scientists have attempted to maximize material removal and tool life while, at the same time, to minimize machining time in order to obtain the best surface finish. As recently as 1959, Olofson [4] reported that a short tool life and low milling production rate could be minimized by a proper selection of feeds, speeds, depth-of-cut, and rigidity of the machine, tool, and workpiece. The findings also showed that machining time, lubrication, cooling methods, and work materials significantly reduce surface roughness.

## 2. Literature Review

Over the years, several researchers have investigated, using both analytical and experimental methods, the contribution of various factors in the milling surface roughness. Baek et al. [5] developed a mathematical model for surface roughness prediction in a face-milling operation by considering the static and dynamic components of the cutting process and verified surface roughness prediction through cutting experiments. Miko and Nowakowski [6] developed a generalized mathematical model of roughness formation for round-nose multi-cutter tools. They reported that tool and workpiece vibration, the cutter run-off, and chips adversely affected the generation of the fine machining surfaces. Agustina et al. [7] performed robot-assisted polishing experiments and reported that the time and frequency domains features of force signal were valuable in estimating the surface roughness. Versaci et al. [8] elaborated on the computing with words (CW) and fuzzy similarity (FS) for the classification of defectiveness of ultrasonic nondestructive evaluation.

Other studies have used Taguchi, fuzzy logic, response surface, and machine learning [9,10] for predicting the machining surface roughness of machine tools. Pimenov et al. [11] used random forest (RF), multilayer perceptron (MLP), regression trees, and radial-based function for the real-time prediction of surface roughness based on the cutting power, machining time, and tool wear data. Yeganefar et al. [12] adopted Support Vector Machine (SVM) to predict optimal surface roughness and cutting power from a number of cutting parameters for machining aluminum alloys.

Researchers have recently been using state-of-the-art Deep Learning (DL) for surface roughness prediction. The performance of ML and DL algorithms depends on the features on which the training and testing are done [13]. Lin et al. [14] experimented with three models: Fast Fourier Transform-Deep Neural Networks (FFT-DNN), Fast-Fourier Transform Long Short-Term Memory Network (FFT-LSTM), and 1D convolutional neural network (1D-CNN). Their result suggested that FFT-LSTM models performed better at higher Ra values, while 1D-CNN was better at predicting lower Ra values. Bhandari and Park [2] proposed a system for evaluating surface roughness employing the distribution of shade on the surface of an object using hybrid CNN-LSTM neural networks. Similarly, Pan et al. [15] used the DL model to establish a relation between the vibration signal and surface roughness.

The bulk of the published papers are based on the energy consumption, machining parameters, vibration parameters, or the force data to predict surface roughness. Although Deshpande et al. [16] addressed the estimation of surface roughness using cutting parameters and cutting force, sound, and vibration in turning operation through a simple regression model, their work focused primarily on finding the coefficient of determination (R^2^).

The present study builds upon the author’s previous work [17], where a transformer-based Deep Learning model was used for predicting roughness classification. However, it lacked benchmarking with other popular deep-learning architectures. This study fills this knowledge gap by benchmarking the legacy DL architectures and the relative new transformer architecture in terms of model parameter size, computational time, and prediction accuracy. The current study is novel in two ways. First, cutting force and machining sound signals were used with cutting-edge DL architectures for roughness classification. Second, four state-of-the-art DL architectures were benchmarked, and classification accuracy was compared for identical training and validation data.

The remainder of the paper is organized as follows: in Section 3, the details of the workpiece material, machines, and experiment design are provided. Cutting force and machining sound data acquisition and data processing are explained in Section 4, which is followed by the presentation of a basic proposed framework of Deep Learning models in Section 5. Section 6 provides details on the model training. The evaluation criteria are defined in Section 7, including results and comparisons, which are followed by conclusions in Section 8. Finally, Section 9 discusses the limitations of the study and future course.

## 3. Machines, Material, and Experimental Design

Aluminum is not only the most abundant metallic element in Earth’s crust, it is also the most widely used nonferrous metal. Notable uses of aluminum include transportation, engineering, construction, and packaging. Fine surface quality is required for aluminum parts to function as desired. In this study, A3003 aluminum plates of 4 mm thickness were used as workpiece materials, since aluminum A3003 is widely used in sheet metal, chemical equipment, and automotive parts because of its good corrosion resistance. Manganese is the primary element in the 3XXX alloy series, enhancing its tensile strength and low-cycle fatigue resistance.

**Figure 2:**
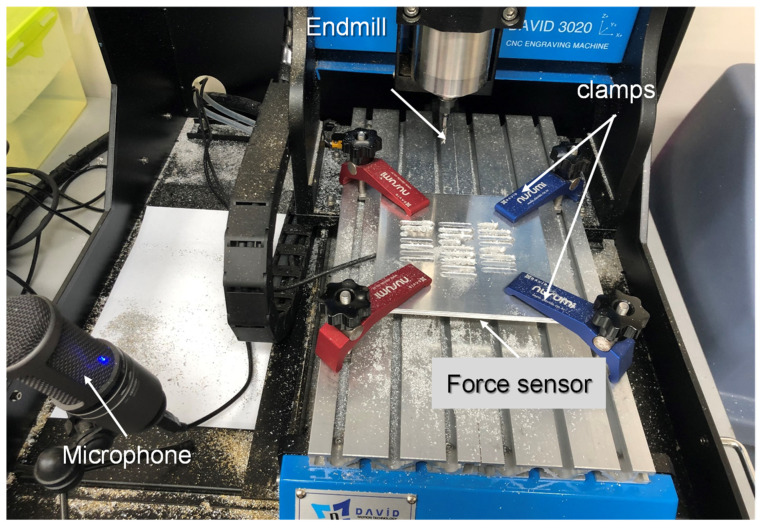
Experimental setup for end-milling operation.

**Figure 3:**
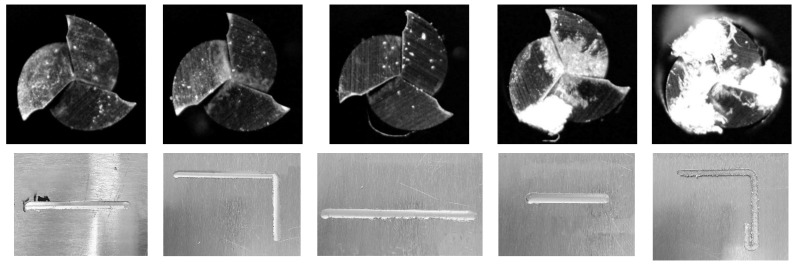
Samples images of end-mill and workpiece after machining experiments (for ∅2 mm end mill).

The end-milling operation was conducted using a DAVID 3020 CNC machine from David Motion Technology. Three flutes of square-end mill of ∅1–∅5 were used in the experiment. An Audio Technica AT2020+ microphone and Cornell University Raven Lite [17] were used for recording the machining sound. Similarly, a capacitive type fore/torque sensor RFT60-HA01 from Robotus Inc. was used for measuring the forces. The end milling experimental setup is shown in Figure 2. The samples of the end-mill condition and workpiece are shown in Figure 3.

Fractional matrix design of experiment (DOE) was used. The details of DOE are provided in [16] and not elaborated here, as the present study extends the previous study.

**Figure 4:**
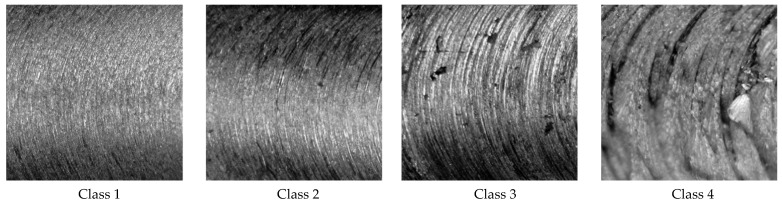
Sample of surface roughness of four different classes of roughness captured by the digital microscope.

As shown in Figure 4, the surface of the machined workpiece has a unique pattern of continuous ridge-and-valley, which is known as surface roughness. For each machining experiment, the surface roughness was measured using the Mitutoyo SJ-210 surface roughness test instrument. Surface roughness is a measure of the total spaced surface irregularities [18]. By convention, 2D roughness parameters are denoted by (capital) *R*, which is followed by additional subscript characters such as *Ra*, *Rq*, and *Rz*. The most widely used surface measurement parameter is *Ra*, which is the arithmetic average of the roughness profile. The formula for calculating *Ra* is given in Equation (1).

(1) $$Ra=\frac{1}{L}\overset{L}{\underset{0}{\int }}\left|z\left(x\right)dx\right|$$

where *L* is the evaluation length, and *Z*(*x*) is the profile height function. Each experiment was classified into four classes based on the surface roughness value (*Ra*), as shown in Table 1.

## 4. Data Acquisition and Preprocessing

### 4.1. Cutting Force Data

**Figure 5:**
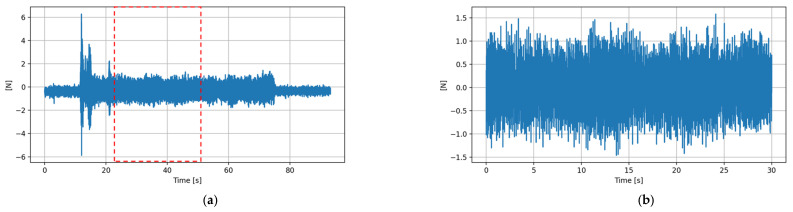
(**a**) A complete force data (*x*-axis) for the 1-min long experiment and (**b**) A cropped force data (10–40 s) of the experiment.

The machining experiments varied from 1 to 5 min. In addition to the experiment length, the force and sound data included pre- and post-experiment data. The data were cleaned to remove excess data by trimming pre- and post-experiment data. In addition, the force data included the clamping force exerted by the jig on the workpiece and the machining force. The clamping force data before starting the experiment was taken as a reference. Since the force sensor measured force in the x, y, and z-directions, $\sum F=F_{x}+F_{y}+F_{z}$ was compared with arbitrary value k, i.e., ∑F>k, where the value of k was found empirically. Force data meeting this condition were marked as the machining experiment start point (0 s), while the machine end time was calculated based on the sampling frequency of the sensors. Figure 5a shows the x-component of force data performed for 1 min with pre and post-experiment data, and Figure 5b shows the x-component of force data cropped for 30 s.

### 4.2. Machining Sound Data

Machining sound was recorded in wav files at a 44.1 kHz sample rate and a 1411 kbps bit rate. Similar to the force data extraction process explained above, the sound data was also extracted from the experimental data using a similar process. Figure 5a shows the complete two-minute experimental sound waveform signal, and Figure 5b shows the cropped sound signal (30 s).

### 4.3. Sound Data Preprocessing

Although the numerical force data can be directly used for DL models, DL models rarely take raw audio directly as input [19] because researchers still doubt whether NN can effectively extract features from the raw input audio signal [20]. The audio is essentially a time-series signal, which is categorized into speech, music, and environmental sounds. The machining sound is an environmental sound that is spread over the whole audible range. Thus, an audio signal needs to go through the feature extraction process, highlighting a signal’s most dominating and discriminating characteristics in a very compact form. The quality of feature extraction decides the DL model performance. The audio features extraction can be sub-categorized into the time domain, the frequency domain, the joint time-frequency domain, and in-depth features.

Time-domain and frequency-domain visualization of audio signals help analyze a few key characteristics of a signal. This information is suitable to predict and analyze similar short signals that have static properties over time. However, with the advent of Deep Learning, deep features are extensively used for analyzing non-stationary sound data employing the windowing technique. Instead of analyzing entire sound data at once, short chunks of a quasi-stationary signal are analyzed with a sliding window function that is zero everywhere except for the region of interest. The window slides over time from the leftmost corner toward the right corner of the plot. The resultant windowed signal is a subset of the original signal passed through the window; the signal is zero for the remainder of the time. This technique is used for noise cancellation, silence reduction, and normalization and is preferred for speaker recognition, music genre classification, and audio analysis.

Generally, when working with audio signals, audio data are trimmed when they are longer while audio clips are padded if they are shorter, to make all the samples the same. Thus, it is easier to work with audio signals with the exact durations; in this study, all the audio signals are exactly 30 s long. Each sound signal was sliced into ten segments (3 s each) to increase the number of samples in each class. Each segment was further sliced into short frames (20–40 ms), assuming that the sound signal does not change over this short time, which is a process widely used in Deep Learning applications. The Python-based audio analysis library ‘librosa’ was used for sound data preprocessing.

When Fast Fourier Transform (FFT) is performed on the audio signal windowed segment, it is called Short-Time Fourier transform (STFT). STFT generates a spectrogram that captures both the time and frequency contents in the signal. To match with the human auditory system, the spectrogram is converted to Mel-scale (from linear scale) using a Mel-scale filter bank, and the generated plot is called a Mel-Spectrogram. The relation between frequency and Mel-frequency is given in Equation (2).

(2) $$M\left(f\right)=1125\ln\left(1+\frac{f}{700}\right)=2595\log_{10}\left(1+\frac{f}{100}\right)$$

**Figure 6:**
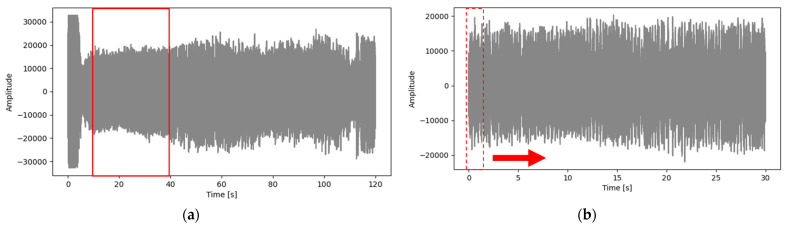
(**a**) A complete sound data of a machining experiment and (**b**) a clipped sound data (10–40 s) used for DL training.

Mel-Spectrograms work well for most audio Deep Learning applications; however, other audio feature extraction methods known as Mel-frequency Cepstral Coefficients (MFCCs) finds application in Automatic Speech Recognition and DL [21]. MFCCs are derived from the cepstral representation of an audio clip based on the discrete cosine transform (DCT) to a Mel-spectrum. In MFCCs, the frequency bands are equally spaced on Mel-scale, which mimics the human auditory system very closely, making MFCCs a key feature in various audio signal processing applications. Another key feature of MFCC is that it has a smaller set of features, capturing the essential quality of the audio signal. The sample of the Mel-Spectrogram and MFCC of machining sound is shown in Figure 6.

### 4.4. Force Data Preprocessing

Force data were logged on the txt file at 200 Hz, one line per sequence for each experiment. Although the force sensor logged force (N) and moment (Nm) data, only force data were used in this study. Although the trimmed force and sound data were each 30 s long, a sampling frequency discrepancy existed between the force and the audio sensors.

**Figure 7:**
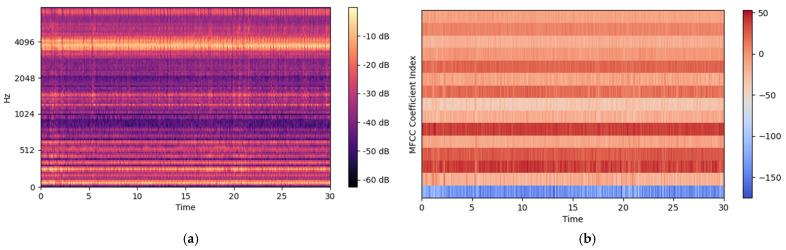
Sample plot of (**a**) Mel-Spectrogram and (**b**) MFCCs of sound data.

**Figure 8:**
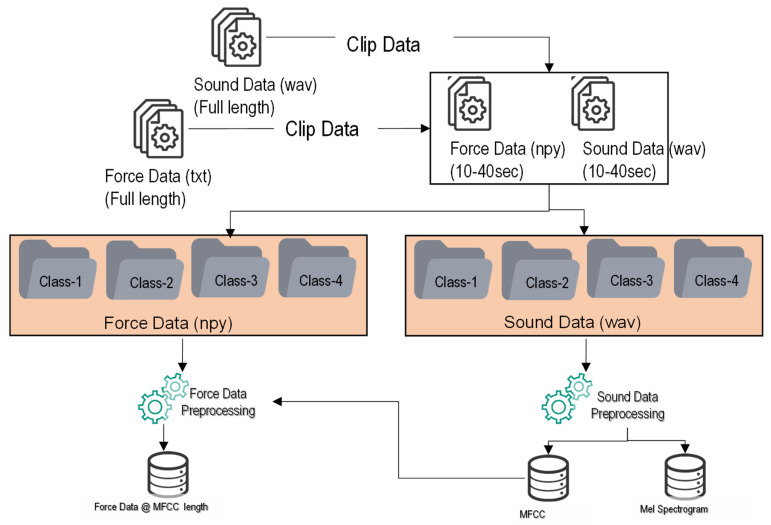
Schema chart of force and sound data processing.

Force data preprocessing followed audio data feature extraction. First, the shape of the MFCC and Mel-Spectrogram was determined and stored in memory. Second, the force data were loaded in the memory, and the length of the force data was determined. Third, the force data length was divided by the length of the MFCC and converted to the nearest integer value, which was used as an index to extract the force data. Finally, the minor discrepancy between the sound and force data was resolved by randomly deleting the excess force data from the force array. The graphical representation of force and sound data processing is shown in Figure 7a,b. The details of sound and force data pre-processing is shown in Figure 8.

## 5. Deep Learning Models

Four types of Deep Learning architectures, viz. simple Multi-Layer Perceptron (MLP), Convolution Neural Network (CNN), Long Short-Term Memory (LSTM), and transformer [22], were analyzed and compared in this study. Each DL model was run twice, firstly with Mel-Spectrogram and secondly with the MFCC feature, and the results were compared. In addition, a preliminary classification using a simple MLP model was conducted to compare the classification accuracy using a machining sound signal only and machining sound and force signals. It was found that using machining sound and force data for the surface roughness classification task produced higher accuracy than using machining sound data only. Thus, all other DL models use both machining sound and force data. The detailed list of DL models used in this study is listed in Table 2.

Altogether, there were four DL models (M1–M4); each DL model had two variants, the first using Mel-spectrogram and the second using MFCCs (except for model M1, which has four variants because of preliminary study). All DL models were trained on 10–40 s machining sound data. For the completeness of this study, the result was benchmarked against the transformer-based model (M4) designed and trained by the author using machining sound and force signals. The results of transformer-based models have been published in [17]. For the sake of completeness, a short description of each Deep Learning architecture is presented below.

### 5.1. Multilayer Perceptron (MLP)

MLP is a relatively simple DL architecture with an input layer, hidden layers, and a final output layer. It is sometimes colloquially referred to as “vanilla” neural networks, especially when the model has a single hidden layer back-propagation network [23]. The signal flows only in one direction, so this architecture is an example of a feedforward neural network (FNN) [24]. For this multi-class classification task, a total of four output neurons (one output neuron per class), a softmax activation function, and a categorical_crossentropy loss function was used. For implementing multiple input data (force and machining sound), the functional Keras API and the ‘concatenate()’ functions were used. The functional Keras API is used to develop a complex model with multiple inputs and numerous modalities that offer ways to create a Deep Learning model with much more flexibility and complexity [2].

### 5.2. Convolution Neural Network (CNN)

CNN is a type of ANN developed based on the human visual nerves [2]. CNN consists of a convolution layer and pooling layer, which makes it different from MLP. Although CNN is primarily used for image recognition and visual tasks, it is not restricted to visual interpretation. CNN has also found application in other tasks such as voice recognition and natural language processing. The convolution and pooling layers have a role in extracting and compressing features from the input data. Equation (3) [24] shows the output computation of a given neuron in the convolution layer.

(3) $$z_{i,j,k}=b_{k}+\overset{f_{h}-1}{\underset{u=0}{\sum }}\overset{f_{w}-1}{\underset{v=0}{\sum }}\overset{f_{n^{\prime }}-1}{\underset{k^{\prime }=0}{\sum }}x_{i^{\prime },j^{\prime },k^{\prime \cdot w_{u,v,k^{\prime },k}}}\;\mathrm{with}\;\left\{\begin{array}{c} i^{\prime }=i\times s_{h}+u\\ j^{\prime }=j\times s_{w}+v \end{array}\right\}$$

where *z_i_*_,*j*,*k*_ is the output of the neuron located in row *i*, column *j* in feature map *k* of the convolution layer (layer *l*), *s_h_* and *s_w_* are the vertical and horizontal strides, *f_h_* and *f_w_* are the height and width of the receptive field, *f*_*n*′_ is the number of feature maps in the previous layer (*l*-1), *x*_*i*′,*j*′,*k*′_ is the output of the neuron located in layer *l*-1, row *i*′, column *j*′ feature map *k*′, *b_k_* is the bias term, and *w_u_*_,*v*,*k*′,*k*_ is the connection weight between any neuron in feature map *k* of the layer *l* [24].

The CNN model used the ‘Conv2D()’ function for convolution operations, ‘MaxPooling()’ for dimensionality reduction, ‘BatchNormalization()’ for accelerating training and improving performance, and ‘Dropout()’ to prevent the model from overfitting.

In this study, the CNN model comprises three convolutional layers with pooling operations; the flattening layers’ outputs are connected to the MLP model used for training machining force to predict the machining surface classification. CNN model architecture is defined in model_configrure() function, which takes two inputs, namely sound data and force data. As in the case of images, CNN architecture expects 4D data (samples, width, breadth, channel). To match the 4D data format, a new axis is added in the 3D format of sound data (samples, samples per segment/hop_length, MFCC coefficients) using Numpy’s numpy.newaxis object as Sound_train[…, np.newaxis]. Similar steps are also performed for validation and testing data (please refer to the source code for the details). As a result of the complex, nonsequential, and wide and deep neural network model, functional Keras API was used. The concatenation layer keras.layers.concatenate() concatenates out from the two branches. The further steps are self-explanatory.

### 5.3. Long Short-Term Memory (LSTM)

**Figure 9:**
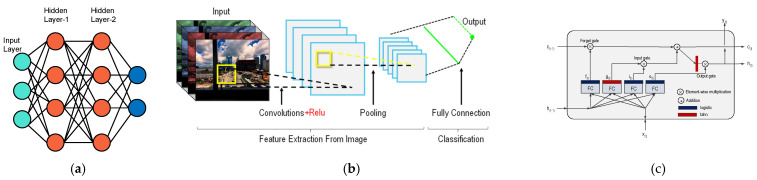
A simplified DL architectures (**a**) MLP, (**b**) CNN, and (**c**) a typical LSTM cell.

LSTM is a Deep Learning architecture that seeks to address the long-term dependency problems of existing Recurrent Neural Networks (RNN) by introducing forget gates. Each gate determines a specific operation, thereby finding the core of the learning data and allowing the model to remember its content longer [25]. Figure 9 shows simple MLP, CNN, and a typical LSTM cell with various labels. Equation (4) summarizes the LSTM cells output at each time-step

(4) i(t)=σ(Wxi XT(t)+WhihT(t−1)+bi)f(t)=σ(Wxf XT(t)+WhfhT(t−1)+bf)o(t)=σ(Wxo XT(t)+WhohT(t−1)+bo)g(t)=tanh(Wxg XT(t)+WhghT(t−1)+bg)c(t)=f(t)⊗c(t−1)+it⊗gty(t)=h(t)=ot⊗tanh(ct)

where *c*_(*t*)_ and *c*_(*t*−1)_ are long-term states at frame *t* and *t* − 1, *h*_(*t*)_ and *h*_(*t*−1)_ are the short-term states at time *t* and (*t* − 1), and *x*_(*t*)_ is the current input vector. Similarly, *W_xi_*, *W_xf_*, *W_xo_*, *W_xg_*, and *W_hi_*, *W_hf_*, *W_ho_*, and *W_hg_* are the weight matrices of each of the four layers to connect to the input vector *x*(*t*)’s previous short-term states *g*_(*t*−1)_, respectively. *b_i_*, *b_f_*, *b_o_*, and *b_g_* are the bias terms for each of the four layers [24].

### 5.4. Transformer Architecture

**Figure 10:**
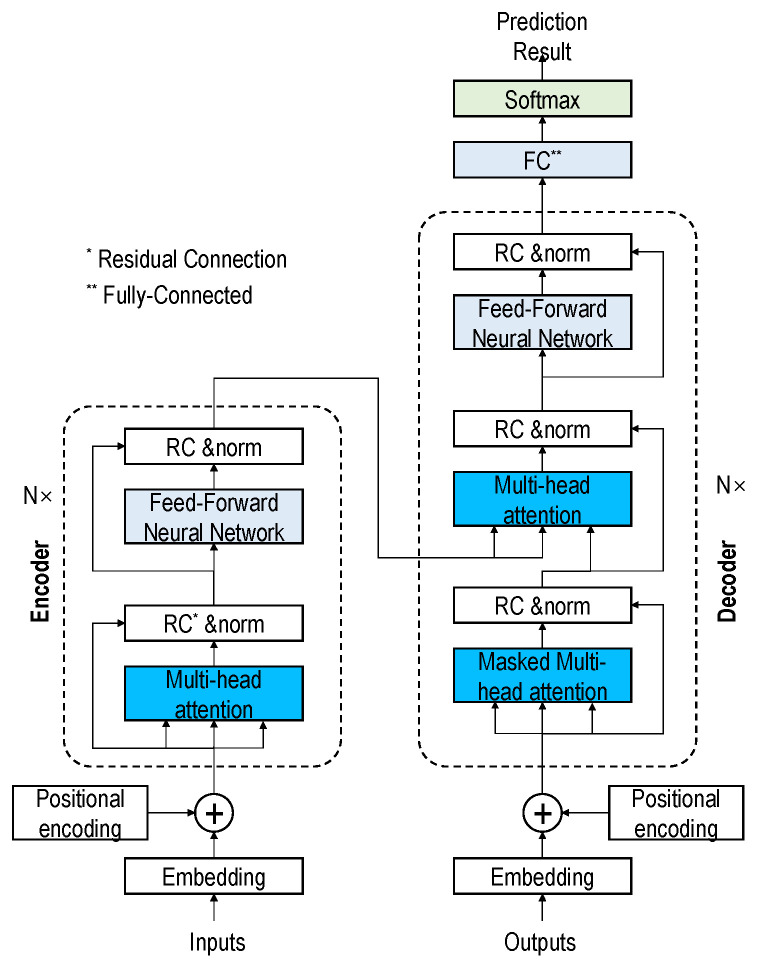
Transformer architecture (redrawn from [22]).

Transformer-based DL architecture has established itself as a new state-of-the-art in the DL world. The transformer-based DL architecture contains a stack of encoder and decoder layers [22] that uses the ‘attention’ mechanism to improve DL models’ performance significantly. Attention mechanisms address the bottleneck problem that arises with the use of a fixed-length encoding vector. The detail of the transformer mechanism is shown in Figure 10.

The encoder maps the input sequence into a continuous abstract representation that holds the input features. Then, the continuous representation is passed onto the decoder block, which generates a single output.

Each encoder layer contains two sub-modules called multi-headed attention and a fully connected feed-forward network with residual connections. The multi-headed attention module has a specific attention mechanism known as self-attention, which allows a model to associate each segment to other segments in the input sample. For this, the input sample is fed into three distinct, fully connected layers to create the ‘query’ (*Q*), ‘key’ (*K*), and ‘value’ (*V*) vectors. The dimensions of *K* and *V* are *d_k_*, and d_v_ respectively. The weight on the values is obtained by computing the dot products of the query with all keys and dividing each by $\sqrt{d_{k}}$. Finally, a softmax function is applied to obtain the weight on the values, as shown in Equation (5).

(5) $$Attention\left(Q,K,V,\right)=softmax\left(\frac{QK^{T}}{\sqrt{d_{K}}}\right)$$

The detailed description of all the components of the transformer architecture is outside the scope of this study. For the detail of the transformer, please refer to the original publication [22].

## 6. Deep Learning Models Training

The cutting force data and machining sound dataset have 160 experimental samples, each 30 s long. Furthermore, each experiment sample was divided into ten segments increasing the number of training samples to 1600 (160 experiment × 10 segments). The preprocessed sound and force data were split into the train–validation–test ratio of 50%–25%–25% during training.

The goal in each DL model considered in this study was to maximize the accuracy and minimize the loss. Model accuracy depends on the choice of the number of training epochs; too many epochs can lead to overfitting, while too few epochs may result in an underfitting model. Therefore, callback methods ‘keras.callbacks.EarlyStopping()’ were used for regularizing and halting the model training at the right time. This allows specifying an arbitrarily large number of training epochs and stopping training once the model performance stops improving. The number of training epochs for all the DL models was set to 500; however, all models reached a minimum validation error close to 100 epochs, upon which training automatically stopped. The parameters were used consistently to compare the DL model architecture. In the case of the transformer-based DL model, the model with Adam optimizer experienced a convergence problem; thus, the optimizer and corresponding learning rate was changed, as shown in Table 3.

Given the stochastic nature of the DL algorithm, evaluation procedure, and numerical precision, it is difficult to get the exact score in DL. Therefore, each model was run five times, and another callback ‘tf.keras.callbacks.CDVLogger()’ method was used to save each epoch result to a CSV file.

## 7. Results and Discussion

The data processing, design of all Deep Learning model architecture, training, validation, and testing codes were written in the Python programming language using TensorFlow’s implementation of the Keras high-level API. The complete code was uploaded to the Github repository and can be found at the following line: https://github.com/thebinayak/benchmark_study.

As mentioned previously, a total of ten models were studied. Each DL model was trained either on Mel-Spectrogram or MFCCs. For consistency, all odd variants (e.g., A, C) were trained on Mel-Spectrogram, while all even variants (e.g., B, D) were trained on MFCCs. The MLP-based DL model has four variants; the first two were trained only on the machining sound data, and the last two were trained with machining sound and cutting force data. Model M1, when only trained on machining sound data, had poor training and validation accuracies, as seen in Table 4. Although the Mel-spectrogram-based training accuracy for M1-A is slightly higher than that for M1-B, the validation accuracies were similar. Compared to all other DL models in this study, the M1-A and M1-B training and validation loss were the lowest; thus, all other models used machining sound and cutting force for DL model training. The training and validation accuracy improved when machining sound and cutting force data were used to train the M1 models (M1-C and M1-D).

Model M2-A took more than 200 epochs to complete the training; despite that, the validation loss was poor amongst all other models. Model M2-A also had the most training parameters.

Transformer-based DL models (M4-A and M4-B) had the lowest number of training parameters, while the validation accuracies were among the highest in all the models. In all the DL models considered in this study, models trained in Mel-Spectrum had more parameters than the models trained in MFCCs because MFCCs are a compressed representation of Mel-Spectrogram.

**Figure 11:**
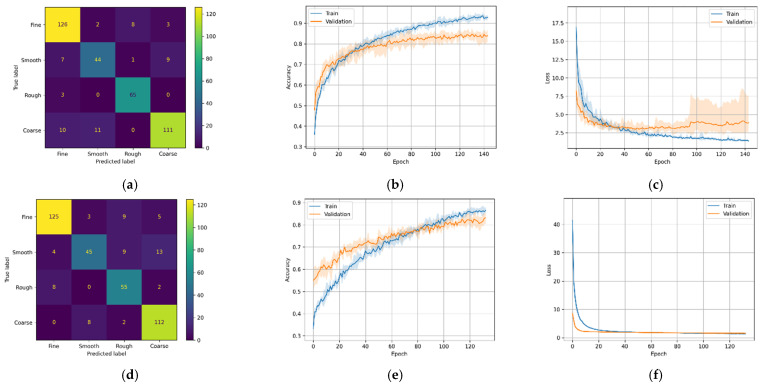

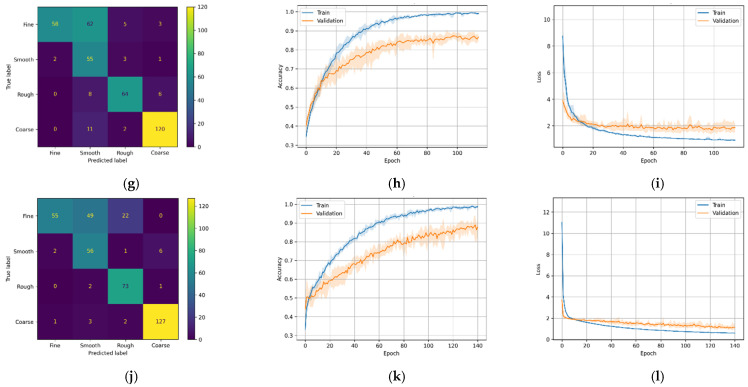
M1 variants confusion matrix, training–validation accuracy, and training–validation loss. (**a**) M1-A confusion matrix; (**b**) M1-A training and validation accuracy; (**c**) M1-A training and validation loss; (**d**) M1-B confusion matrix; (**e**) M1-B training and validation accuracy; (**f**) M1-B training and validation loss; (**g**) M1-C confusion matrix; (**h**) M1-C training and validation accuracy; (**i**) M1-C training and validation loss; (**j**) M1-D confusion matrix; (**k**) M1-D training and validation accuracy; (**l**) M1-D training and validation loss.

**Figure 12:**
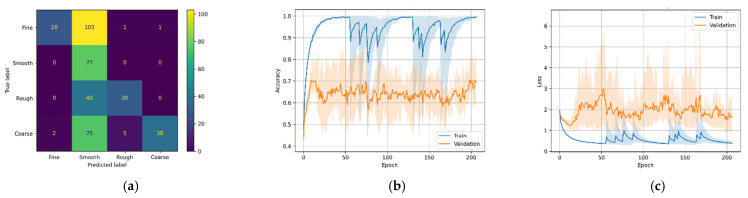

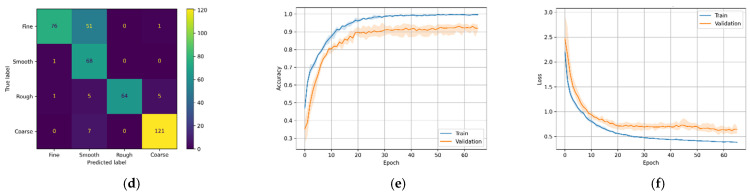
M2 variants confusion matrix, training–validation accuracy, and training–validation loss. (**a**) M2-A confusion matrix; (**b**) M2-A training and validation accuracy; (**c**) M2-A training and validation loss; (**d**) M2-B confusion matrix; (**e**) M2-B training and validation accuracy; (**f**) M2-B training and validation loss.

**Figure 13:**
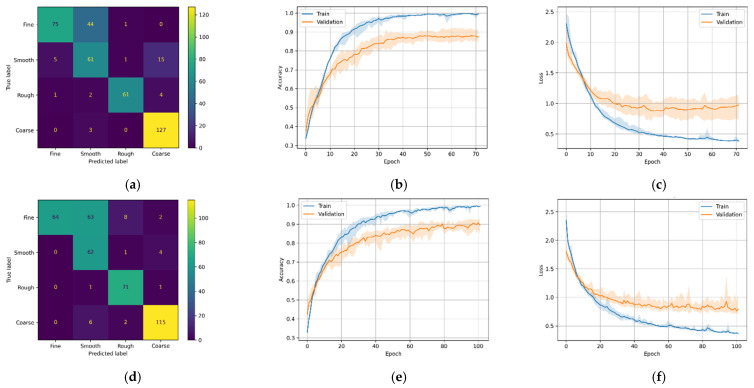
M3 variants confusion matrix, training–validation accuracy, and training–validation loss. (**a**) M3-A confusion matrix; (**b**) M3-A training and validation accuracy; (**c**) M3-A training and validation loss; (**d**) M3-A confusion matrix; (**e**) M3-B training and validation accuracy; (**f**) M3-B training and validation loss.

**Figure 14:**
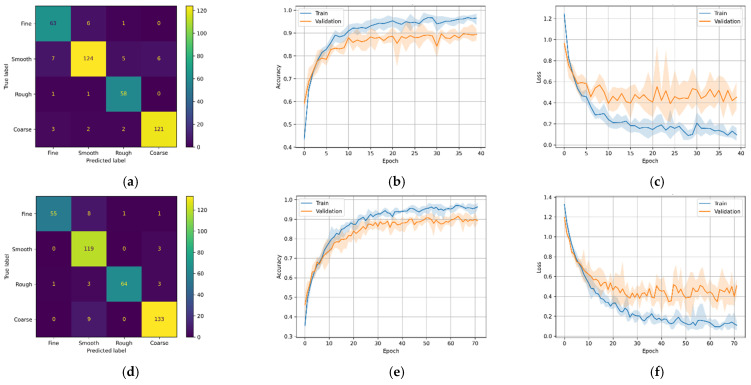
M4 variants confusion matrix, training–validation accuracy, and training–validation loss. (**a**) M4-A confusion matrix; (**b**) M4-A training and validation accuracy; (**c**) M4-A training and validation loss; (**d**) M4_B confusion matrix; (**e**) M4-B training and validation accuracy; (**f**) M4-B training and validation loss.

As a result of the stochastic nature of the DL algorithm, the results of the DL models change slightly in every run. Therefore, each model was run five times, and the average score was plotted in Figure 11, Figure 12, Figure 13 and Figure 14 (second and third column). The ‘matplotlib.pyplot.fill_between()’ function was used to generate a filled plot, using the upper and lower bounds of training accuracy and validation accuracy, as shown in the figures below.

All the trained DL models were further used for inference purpose to predict the unseen testing data (25% of 1600). For most DL architectures, the inference results showed comparable model accuracy and loss results compared to the validation accuracy and loss, confirming that the models were generalized well. It is difficult for the multi-class classification problem to make sense of the inference results only by comparing inference accuracies.

To overcome this problem, confusion matrices were plotted for the inference results to visually observe the prediction accuracy of the models using ‘sklearn.metrics.confusion_matrix()’ method. The confusion matrix provides an overall idea of how accurate the models are at inferring on the test data. The confusion matrices can be seen in the first column of Figure 11, Figure 12, Figure 13 and Figure 14. As seen in the figures, the predicted labels are on the *x*-axis, and the true labels are on the *y*-axis. Generally, the confusion is read from the top left to the bottom right diagonally; the values in the diagonal are the correct predictions.

For M1-A, Figure 11a shows 346 correct predictions out of 400 test samples (or 86.5%). Similarly, Figure 11d,g,j shows 337 (or 84.2%), 297 (74.2%), and 311 (77.7%) correct predictions, respectively. The figures illustrate that for the first two cases (using only machining sound data), the confusion matrices have similar distribution; both of the models were good at classifying course and fine classes.

However, for the latter two cases, although the validation accuracy increases significantly compared to the first two, the inference accuracy drops slightly, as seen in the confusion matrix. Both models (M1-C and M1-D) better classify course roughness while they had difficulty classifying the fine and smooth classes.

In the case of model M2-A, which uses CNN architecture with Mel-Spectrogram, the training accuracy steadily increased for the first few epochs, after which the training and validation accuracy and training and validation loss had an erratic pattern, as seen in Figure 12b,c, respectively. As a result of this erratic pattern, model M2-A took more epochs (>200 epochs) to train but failed to improve the validation accuracies satisfactorily. While the M2-B variant (with MFCCs) shows a steady increase in training and validation accuracies, no abnormalities can be seen in the M2-B training and validation loss. With these results, it can be inferred that in the case of CNN architecture with fewer training data, MFCC-based feature extraction performs better than Mel-Spectrogram. The confusion matrix Figure 12d has excellent results, with most of the classes predicted accurately while struggling to classify the fine class 329/400 (or 82.2%).

Similar to the CNN models above, the LSTM models have similar patterns. Figure 13a,b show that the models were good at predicting course, rough, and smooth roughness classes but had difficulty in correctly predicting fine classes. As seen in the confusion matrices, model M3-A correctly predicted 324 (or 81.0%), and M3-B correctly predicted 312 (or 78.0%) out of 400.

The best-performing model in this study is the transformer-based DL model (M4). It can be seen that the transformer-based DL model trained on MFCC data had higher validation accuracy than the model trained on the Mel-Spectrogram data. This is evident because MFCC is a compressed, decorrelated version of the Mel-Spectrum. A previous study [26] showed that MFCCs often perform better when limited data are available. The confusion matrices in Figure 14a,d show that model M4-A correctly predicted 366 (or 91.5%) and model M4-B correctly predicted 371 (or 92.7%) out of 400 test samples.

## 8. Conclusions

This study is a continuation of an extensive study on implementing various DL architectures to predict surface roughness in real time. The machining surface roughness was categorized into four classes viz. fine, smooth, rough, and coarse. There have been a concern and need for comparative study on the prediction accuracy of many popular deep-learning models. This study benchmarked the four most popular DL architectures, MLP, CNN, LSTM, and transformer using machining sound and cutting force data. Since raw sound data are seldom used in DL models, two of the most widely used audio feature extraction techniques, Mel-Spectrogram and MFCCs, were used in each model to compare the suitability and performances of the models.

Complex functional DL models were designed using Keras functional API for multiple inputs (machining sound and cutting force). It was found that employing machining sound and cutting force data yielded better training and validation accuracy than just using machining sound data.

One of the most critical requirements of DL models is to be trained in the shortest possible time. MLP-based DL models took more than 100 epochs to train and automatically stopped after the model performance saturated. CNN and LSTM-based models took less than 100 epochs to train, except for model M2-A, which performed poorly in Mel-Spectrogram data. The most efficient models were transformer-based DL models, which took less than 70 epochs and had the lowest number of training parameters.

In the case of classification accuracy, the most accurate DL models were transformer-based DL models. The confusion matrix suggested that M4-A and M4-B have prediction accuracies close to 92%. No other DL model considered in this study was comparable to transformer-based DL models. It is proposed that this benchmarking study could be used to select the DL models to be used in predicting the machining surface roughness in real time with high accuracy in real-time machining operation. Since the transformer-based architecture has the least parameters, it can also be used in edge computing, making the implementation even cheaper and more portable.

## 9. Limitations and Future Study

This study can be considered an essential milestone in machining surface roughness prediction using DL models. The current study sheds light on the accuracy and suitability of various Deep Learning architectures for force and sound data used in machining operations. However, the author would like to point out a few potential future study areas. In the present study, the machining experiments with each roughness class were small; more extensive experiments data with an extensive range of end-mill diameters will undoubtedly be helpful for DL training. Furthermore, this study was conducted on the aluminum plate; however, engineering materials differ in their physical and chemical properties, so more studies are needed for a multitude of materials. In addition, the current study considers only four roughness classes; however, to match the ISO standards, more roughness classes must be implemented. This can be achieved by collaboration between research institutes and the manufacturing industry.

## Figures and Tables

**Table 1:** Machined surface categories based on the *Ra* values.

Class	*Ra* Range (μm)	ISO Grade Number
Class 1	0–1	N1–N6
Class 2	1–2	N7
Class 3	2–4	N8
Class 4	>4	N9+

**Table 2:** DNN architectures and its variants.

Model	Variant	DNN Architecture	Signal (s)			Data Duration	Audio Feature Extraction
M1	A	MLP	Machining sound only			10–40 s	Mel-Spectrogram
	B						MFCC
	C		Machining sound	+	Force		Mel-Spectrogram
	D						MFCC
M2	A	CNN	Machining sound	+	Force		Mel-Spectrogram
	B						MFCC
M3	A	RNN-LSTM	Machining sound	+	Force		Mel-Spectrogram
	B						MFCC
M4	A	Transformer	Machining sound	+	Force		Mel-Spectrogram
	B						MFCC

**Table 3:** Parameters used for compiling the DL models.

	M1, M2, M3	M4
Loss Function	categorical_crossentropy	categorical_crossentropy
Optimizer	Adam	RAdamOptimizer
Learning Rate	0.0001	0.01
metrics	accuracy	accuracy

**Table 4:** Average epoch, training accuracy, and validation accuracy for each model for comparison.

Models	Signal Used	Variant	Audio Feature Extraction	Max Epochs	Training Accuracy	Val Accuracy	Total Parameters
M1	Sound Only	A	Mel-Spectrogram	144	0.928	0.839	8,934,468
		B	MFCC	133	0.865	0.831	1,146,948
	Sound + Force	C	Mel-Spectrogram	115	0.990	0.866	8,887,940
		D	MFCC	140	0.986	0.877	8,828,036
M2	Sound + Force	A	Mel-Spectrogram	207	0.996	0.692	12,292,740
	Sound + Force	B	MFCC	65	0.996	0.919	4,919,940
M3	Sound + Force	A	Mel-Spectrogram	72	0.995	0.873	4,532,612
	Sound + Force	B	MFCC	102	0.994	0.895	4,502,660
M4	Sound + Force	A	Mel-Spectrogram	40	0.966	0.894	299,445
	Sound + Force	B	MFCC	71	0.963	0.892	34,674

## Data Availability

The complete code, including data creation, data preprocessing, model design, and validation codes, is uploaded to the Github repository and found at the following link https://github.com/thebinayak/benchmark_study.
